# MiR-539 inhibits proliferation and migration of triple-negative breast cancer cells by down-regulating LAMA4 expression

**DOI:** 10.1186/s12935-018-0512-4

**Published:** 2018-01-30

**Authors:** Zhi-Xue Yang, Bo Zhang, Jinrong Wei, Guo-Qin Jiang, Yan-Lin Wu, Bing-Jing Leng, Chun-Gen Xing

**Affiliations:** 1 Department of General Surgery, The Second Affiliated Hospital, Soochow University, Suzhou, 215004 Jiangsu China; 2 Department of Radiology, The Second Affiliated Hospital, Soochow University, Suzhou, 215004 Jiangsu China

**Keywords:** miR-539, LAMA4, Proliferation, Migration, TNBC

## Abstract

**Background:**

Recent studies have shown that laminin subunit alpha 4 (LAMA4) plays an important role in carcinogenesis. However, its molecular biological function in triple-negative breast cancer (TNBC) has not been entirely clarified. This study investigated the expression of LAMA4 in TNBC and its effect on cell proliferation, migration and invasion. Furthermore, we also identified the potential miRNA directly targeting LAMA4.

**Methods:**

Western blot, Real-time quantitative PCR (qPCR) and immunohistochemical staining (IHC) were used to detect the expression of LAMA4 in TNBC. The effects of LAMA4 on TNBC cell proliferation, migration and invasion were also explored in vitro. The potential miRNA that targets LAMA4 was determined by dual luciferase reporter assay and verified by qPCR and western blot analysis.

**Results:**

Our study showed LAMA4 mRNA (p = 0.001) and protein (p = 0.005) expression in TNBC tissue samples were elevated compared with adjacent normal tissue samples, and LAMA4 was mainly expressed in the cytoplasm of breast carcinoma cells. Knockdown of LAMA4 inhibited TNBC cell proliferation, migration and invasion in vitro. Moreover, further study revealed that LAMA4 was a putative target of miR-539, and miR-539 negatively regulated LAMA4 expression by directly targeting its 3′-UTR.

**Conclusions:**

Our study suggested that miR-539 suppressed the expression of LAMA4. LAMA4 plays an important role in tumor progression and may be an important target in treatment of TNBC.

## Background

Breast cancer is the most common type of cancer in women worldwide and its incidence is increasing [1, 2]. Importantly, triple-negative breast cancer (TNBC) is the most invasive and aggressive one among the breast cancer subtypes [3], lacking expression of α-estrogen, progesterone and HER2(erbB2)receptors and characterized by high mitotic rate, increased lymphocytic infiltrate,high grade and large tumor size [4, 5]. The lack of targeted therapies and the poor prognosis of patients with TNBC have fostered a major effort to discover actionable molecular targets to treat patients with these tumors, but the clinical and molecular heterogeneity within TNBC subtype makes the treatment of these patients even more challenging [5, 6]. It is generally recognized that the improvement of prognosis, prediction of response to treatment, and development of novel effective therapeutic approaches for TNBC is urging, which may largely depend on the introduction into clinical practice of novel specific markers involved in the development of TNBC. Therefore, identifying biological markers of TNBC progression could be meaningful for the prevention of TNBC and providing new therapeutic strategies for the disease.

As a key molecule of the extracellular matrix, laminins provide a delicate microenvironment for cell functions [7], more than 15 laminin isoforms are known to date and the expression of specific isoforms may change in certain pathological conditions [8, 9]. Among these, Laminin a4 (LAMA4) is widely distributed in developing and adult human tissues, and mainly localized to mesenchyme-derived tissues [10]. It has been reported that LAMA4 plays a role in formation and function of endothelium, transmigration of inflammatory cells through endothelium and invasion of certain tumors [11–13]. Besides, LAMA4 is thought to play a role in cell migration, wound healing and angiogenesis [13, 14]. So we are curious about its role in TNBC and think it may be important for TNBC progress.

MicroRNAs (miRNAs) are a class of endogenous noncoding single-stranded RNAs, which regulate amounts of protein expressed from coding RNAs by translational repression or by cleavage of the target mRNA due to base pairing with the 3′-untranslated region (UTR) [15]. MiRNAs were found to be linked to essential physiologic processes such as proliferation, differentiation and apoptosis, and to several diseases including cancer [16, 17]. In recent years, the fundamental role of miRNAs in cancer progression and metastasis is beginning to be elucidated. MiRNA expression studies, especially large-scale profiling, have provided evidence that the aberrant expression of miRNA is associated with human breast cancer [18, 19]. These finds will aid in early diagnosis using these miRNAs as markers, and functional studies of specific miRNAs, determining their targets, function and regulation.

The objective of this article is to explore novel specific markers involved in the development of TNBC and design therapies that target them so as to prevent systemic cancer.

## Methods

### Clinical specimens

TNBC and adjacent normal breast tissues were anonymously collected from The Second Affiliated Hospital of Soochow University (n = 40). Written informed consent was obtained from each patient. All of the procedures performed in this study were approved by the Ethics Committee of The Second Affiliated Hospital of Soochow University and we have obtained consent to publish from the patients provided the tissues.

### Immunohistochemistry assay

Immunohistochemical analysis was done to study the expression of LAMA4 in TNBC tissues and the adjacent normal breast tissues. Formalin-fixed, paraffin-embedded tissue was freshly cut (3 mm). Sections were deparaffinized in xylene and re-hydrated in a graded series of ethanol. Endogenous peroxidase activity was blocked with 3% hydrogen peroxide. Antigen retrieval was achieved by boiling slides in 10 mM citrate buffer (pH 6.0) for 20 min. 10% non-immune goat serum was used to block nonspecific binding. Sections were then incubated in a moist chamber with primary rabbit anti-human LAMA4 antibody (1:200; Cat. No. 10465-1-AP, Proteintech, USA) for 30 min at room temperature, followed by a secondary antibody (peroxidase labeled polymer conjugated to goat anti-rabbit immunoglobulin) for 30 min (DakoCytomation, Denmark). Rabbit serum was used as negative control. All slides were developed with diaminobenzidine (DAB). Slides were counterstained with hematoxylin, dehydrated through a graded series of ethanol, immersed in xylene and mounted.

### Cell culture

TNBC cell line BT-549 and Human Embryonic Kidney 293 (HEK-293T) cells were purchased from Chinese Academy of Science Cell Bank (Shanghai, People’s Republic of China). BT-549 and HEK-293T cells were cultured in Dulbecco’s Modified Eagle’s Medium (DMEM; HyClone, Logan, UT, USA) with 10% fetal bovine serum (FBS). All media contained 1% penicillin/streptomycin. All cell lines were incubated at 37 °C with 5% CO_2_.

### Transfection

For miRNA-539-overexpression, cells were seeded at ∼ 70% confluence into six-well plate and transiently transfected with miR-539 mimic or a scrambled miRNA to a final concentration of 50 nM using 5 μL Lipofectamine 2000 transfection reagent (Thermo Fisher Scientific, Waltham, MA, USA, USA) following the manufacturer’s instruction.

### Adenovirus infection

On the day before virus infection, BT-549 cells were plated in each well of six-well plates. When the cells reached approximately 70% confluence, the culture medium was aspirated and the cell monolayer was washed with prewarmed sterile phosphate-buffed saline (PBS). Cells were incubated with indicated virus 5 μL LV (Lentiviral vector–derived)-LAMA4-shRNAs (short hairpin RNA) or LV-NC (negative control), IO:10^9^, GenePharma Co., Ltd, Shanghai, People’s Republic of China), respectively. After adsorption for 4 h, 2 ml of fresh growth medium was added and cells were placed in the incubator for additional 48 h. The cells analysis and other experiments were performed.

### Western blotting

Western blotting was performed as described in our previous study [20]. Briefly, cells were harvested and rinsed with PBS. Tumor tissues, adjacent normal breast tissues and cell extracts were prepared using lysis buffer (containing 8 M urea, 10% SDS (sodium dodecyl sulfate), 1 M DTT and protease inhibitors) and centrifuged at 12,000*g* at 4 °C. Total protein concentration was measured using the BCA (bicinchoninic acid) assay. Cellular extracts containing 30 μg total protein were electrophoresed on 10% SDS-PAGE gels and then transferred onto polyvinylidene difluoride membranes (Invitrogen). The membranes were incubated for 2 h in blocking solution containing 5% non-fat dry milk to inhibit non-specific binding, then incubated with primary anti-LAMA4 (1:2000; proteintech, USA) and anti-β-actin FLAG (1:5000; Abcam, Cambridge, MA, USA) antibodies for 2 h. After several washes in PBS, the membranes were incubated with HRP-conjugated secondary antibodies (1:4000; Abcam, Cambridge, United Kingdom). The blots were developed using an ECL chemiluminescent kit (Beyotime, Haimen, China), and exposed to X-ray film for 30 s–2 min. The densities of protein bands were analyzed using PDQuest software version 7.2.0 (Bio-Rad Laboratories, Inc., Hercules, CA, USA). The expression of LAMA4 protein was normalized to β-actin.

### Quantitative PCR

Total cellular or tissues’ RNA was isolated using TRIzol (Invitrogen) according to the manufacturer’s instructions. For reverse transcription (RT)-PCR, 5 μg of total RNA per sample was reverse transcribed using the Reverse Transcription Reaction Kit (Fermentas, St. Leon-Rot, Germany) according to the manufacturer’s instructions. The cDNA (1 μl) was amplified by PCR (pre-denaturation step at 95 °C for 5 min; followed by 40 cycles of 95 °C for 30 s, 60 °C for 30 s, and 72 °C for 30 s; then 72 °C for 10 min). The primers were as follows: LAMA4, 5′-AAG CAG AGT CTC TGT GAT GGCAG-3′ and 5′-GTC CTG TTC AAC TCG ATG AAAGC-3′;GAPDH, 5′-TCCTGTGGCATCCACGAAACT-3′ and 5′-GAAGCATTTGCGGTGGACGAT-3′. The final, normalized results were calculated by dividing the relative transcript levels of the target genes by the relative transcript levels of GAPDH.

### MTT assay

BT-549 cells (5.0 × 103/well) were seeded into five 96-well culture plates (6-parallel wells/group). On each day, 200 μL MTT (5 mg/mL) was added to each well, and the cells were incubated for additional 4 h at 37 °C. Then the reaction was stopped by lysing the cells with 150 μL DMSO for 5 min. Optical densities were determined on a Versamax microplate reader (Molecular Devices, Sunnyvale, CA) at 490 nm.

### Wound healing assay

BT-549 cells were seeded in 6-well dishes at a density of 1 × 106/well and treated with LV-NC or LV-LAMA4-shRNA3. After 24 and 48 h of incubation, a scratch in the cell monolayer was made using a sterile micropipette tip. Cells were washed twice with fresh media, and images were captured using an inverted microscope (IX71; Olympus, Center Valley, PA, USA) at 24 and 48 h after scratching. The rate of wound healing was estimated by measuring the distance between the borders of the wound.

### Matrigel invasion assay

Invasion assays were performed by using the same transwell chamber with growth factor-reduced Matrigel. Briefly, 1 × 10^5^ cells infected with LV- NC or LV-LAMA4-shRNA3 per well were seeded onto Matrigel-coated inserts and allowed to invade for 48 h. Cells remaining above the insert membrane were removed with a cotton swab, and cells that invaded through the Matrigel were fixed in 25% methanol. After washing in cold 1 × PBS, the cells were stained with 0.1% crystal violet in 25% methanol. The inserts were washed three times with 1 × PBS and then air-dried. The numbers of invaded cells on the representative sections were counted using an inverted microscope (IX71; Olympus) at 10× magnification. Five fields were counted per filter in each group; the number of invaded cells for each sample represents the average of triplicate wells over three experiments.

### Luciferase reporter assay

The dual luciferase experiment was performed in Human Embryonic Kidney 293 (HEK-293T) cells. A 1507 bp fragment of the LAMA4 3′-UTR (corresponding to the positions of 5847–7355 of the NM_002290.4) was amplified by PCR using the cDNA of HEK-293T cells as a template and cloned into the pGL3-basic vector (Promega, Madison, WI) to generate pGL3- LAMA4-3′-UTR plasmid. Mutation of the putative miR-539 target sequences within the 3′ UTR of LAMA4 in the pGL3-LAMA4-3′-UTR plasmid was generated using the QuikChange Site-Directed Mutagenesis kit (Stratagene). Plasmid DNA was subsequently isolated from recombinant colonies and sequenced to ensure the authenticity and direction of the inserted *LAMA4* 3′UTR. HEK-293T cells were cultured in six-well plates at 80% confluence and cotransfected with 2 μg pGL3-LAMA4-3′-UTR plasmid or pGL3-LAMA4-3′-UTR-mutant plasmid together with 70 nM miR-539 mimic or a scrambled miRNA using 4 μL Lipofectamine 2000 reagent according to the manufacturer’s protocol. 10 ng pRL-TK vector (Promega, USA) was co-transfected as internal control for normalization of the transfection efficiency. Cells were lysed and assayed for luciferase activity at 48 h after transfection using a Dual-Luciferase Assay kit (E1910; Promega). Changes in the expression of Renilla luciferase were normalized relative to Firefly luciferase.

### Statistical analyses

Statistical analyses were performed using SPSS version 21.0 (SPSS Inc., Chicago, IL, USA). All data were presented as the mean ± standard deviation. The Student t test was used to determine significance of changes between two groups. Mann–Whitney test following Friedman ANOVA was used for multiple comparisons where appropriate. Normality was verified for all data. Values of p < 0.05 were considered to be statistically significant.

## Results

### Expression of LAMA4 is increased at both mRNA and protein level in TNBC tissues

**Fig. 1:**
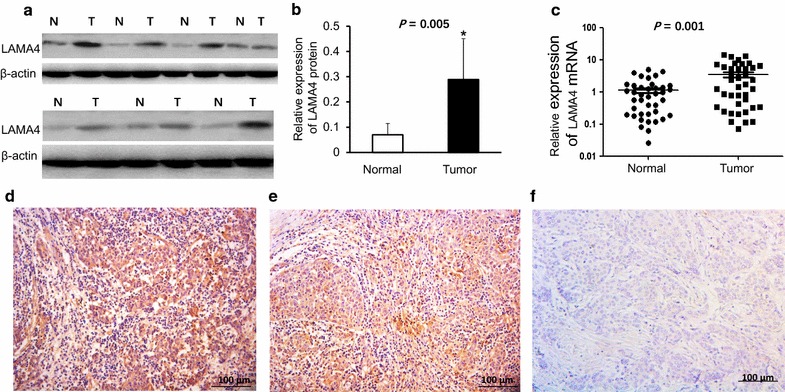
Expression of LAMA4 is increased at both protein and mRNA level in TNBC tissues. **a** The representative western blot results in 7 cases are shown. **b** The protein level of LAMA4 was higher than in adjacent normal tissues, p = 0.005, n = 40, Student’ t test. **c** The mRNA level of LAMA4 in TNBC was significantly higher compared with adjacent normal tissues, p = 0.001, N = 40, Student t test. **d**–**f** LAMA4 was mainly expressed in the cytoplasm of breast carcinoma cells, **d** high expression; **e** low expression; **f** negative expression

To determine whether LAMA4 plays a pivotal role in TNBC, western blotting assays and quantitative PCR were performed in TNBC and adjacent normal tissues. The representative western blot results in 7 cases are shown in Fig. 1a. The average LAMA4 protein level in 40 TNBC tissues was significantly higher than that in corresponding adjacent normal tissues (Fig. 1b, p = 0.005, n = 40 for each group, Student t test). The mRNA level of LAMA4 in TNBC was significantly higher compared with adjacent normal tissues (Fig. 1c, p = 0.001, n = 40, Student’ t test). Immunohistochemical analysis was performed to study its expression and location, the result showed LAMA4 was mainly expressed in the cytoplasm of breast carcinoma cells, 32 patients of the samples had a high expression of the LAMA4 (Fig. 1d) while the other 8 patients have a low expression (Fig. 1e). In adjacent normal tissues, there were negative expression of LAMA4 (Fig. 1f). Taken together, these data suggest that LAMA4 was mostly overexpressed in TNBC tissues compared with adjacent normal tissues.

### Lentiviral vector–derived shRNA approach against LAMA4 suppresses LAMA4 expression both at protein and mRNA level

**Fig. 2:**
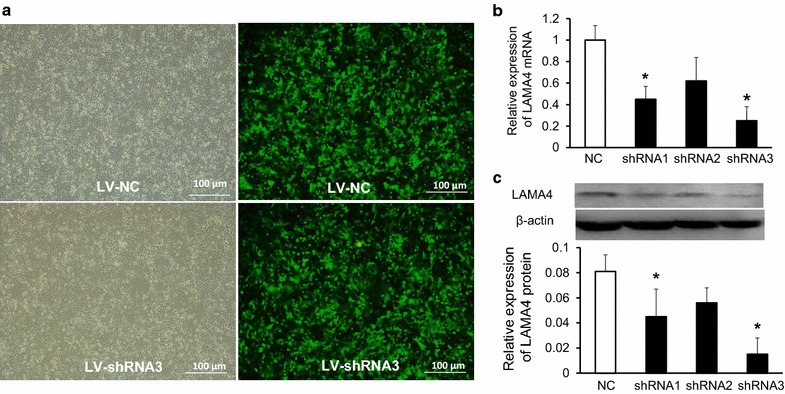
Lentiviral vector–derived shRNA approach against LAMA4 suppresses LAMA4 expression both at protein and mRNA level. **a** The images of tumor cells before and after infection with LV-LAMA4-shRNAs and LV-NC. **b** LV-LAMA4-shRNA1 and LV-LAMA4-shRNA3 could effectively silenced the expression of LAMA4 in mRNA level, n = 4, *p < 0.05, Mann–Whitney test following Friedman ANOVA. **c** LV-LAMA4-shRNA1 and LV-LAMA4-shRNA3 could effectively silenced the expression of LAMA4 at protein level (n = 4, *p < 0.05, Mann–Whitney test following Friedman ANOVA)

To further determine whether LAMA4 is involved in the regulation of development of TNBC, we have developed a highly efficient method of lentivirus-mediated delivery of siRNA targeting LAMA4 for gene silencing. We designed three LV-LAMA4-shRNAs using different sequences, LV-LAMA4-shRNA1 (AAGTCTCCATGATGTTTGATG) LV-LAMA4-shRNA2 (AAGTGCGGCTAGATTCTCAGA) and LV-LAMA4-shRNA3 (AAGGCCTTCACGTCTCTGAGC), then we transfected BT-549 cells with the LV-LAMA4-shRNAs or LV-NC. As shown in Fig. 2a, the infection efficiency could reach more than 90 percent both in LV-LAMA4-shRNAs and LV-NC groups. Then western blotting assays and quantitative RT-PCR were performed and found LV-LAMA4-shRNA3 had a highest efficiency of gene silence. After 48 h after infection, the protein levels were detected. The data showed that LV-LAMA4-shRNA1 and LV-LAMA4-shRNA3 could effectively silenced the expression of LAMA4 both in protein and mRNA level but not the LAMA4-shRNA2, and LV-LAMA4-shRNA3 had a better gene silence efficiency (n = 4 for each group, *p < 0.05, Mann–Whitney test following Friedman ANOVA).

### Silence of LAMA4 inhibits TNBC cancer cell proliferation, decreased cell migration and slowed cell invasion in vitro

**Fig. 3:**
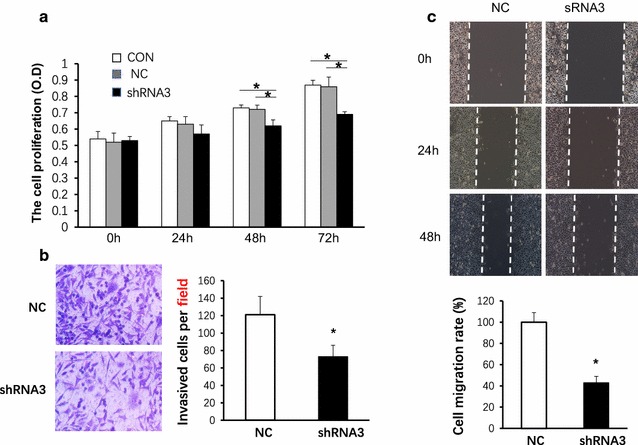
Silence of LAMA4 inhibits TNBC cancer cell proliferation, decreased cell migration and slowed invasion in vitro. **a** The cell proliferation of LV-LAMA4-shRNA3 infected cells was inhibited after 48 h even to 72 h later, but neither at 24 h nor before compared with the control group cells and LV-NC cells. *p < 0.05, n = 3, Mann–Whitney test following Friedman ANOVA). **b** LV-LAMA4-shRNA3 significantly inhibited the invasive ability of BT-549 cells, *p < 0.05, N = 4 Student t test. **c** LV-LAMA4-shRNA3 could inhibited the cell migration, *p < 0.05, N = 4, Student t test

The proliferation of BT-549 cells was measured by MTT assay. After incubated for the indicated time, the BT-549 cells were infected by LV-NC or LV-LAMA4-shRNA3. The results showed that the cell proliferation of LV-LAMA4-shRNA3 infected cells were inhibited at 48 h even lasted to 72 h, but neither at 24 h nor before compared with the control group cells or LV-NC cells (Fig. 3a, *p < 0.05, n = 3 for each group, Mann–Whitney test following Friedman ANOVA). Cell migration was evaluated using wound healing and transwell assays. Results showed that LV-LAMA4-shRNA3 inhibited the invasive ability of BT-549 cells by approximately 38.8%, as the Invasive cells per field in LV-LAMA4-siRNA3 group was 78 ± 13 while it was 121 ± 22 in LV-NC group (Fig. 3b, *p < 0.05, N = 4 for each group, Student t test). Cell mobility was detected also by the wound healing assay. Cells were grown to confluence in 6-well plates, wounded, and transfected with LV-NC and LV-LAMA4-shRNA3 respectively. Scratch closure was monitored for 48 h; microscopic images taken at 0, 24 and 48 h post-scratching are shown. At 24 and 48 h after wounding, the healing ability of LV-LAMA4-shRNA3-infected BT-549 cells significantly lagged behind the LV-NC -infected BT-549 cells. Images were captured at a magnification of × 10, and the columns represent the means ± SEMs of four independent experiments at 48 h. The results showed that LV-LAMA4-siRNA3 could inhibit the cell migration rate to 43% (Fig. 3c, *p < 0.05, N = 4 for each group, Student t test).

### MiR-539 was decreased in TNBC tissues and has an inverse correlation with the expression with LAMA4

**Fig. 4:**
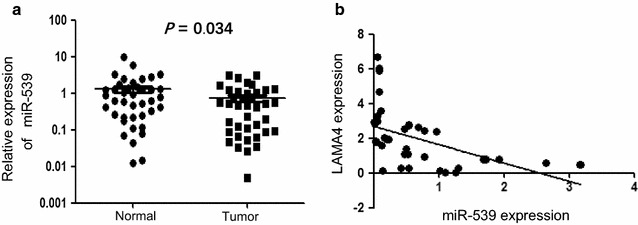
MiR-539 was decreased in TNBC tissues and has an inverse correlation with the expression with LAMA4. **a** The expression of miRNA-539 was decreased in TNBC tissues compared with the adjacent normal tissues p = 0.034, n = 40, Mann–Whitney test following Friedman ANOVA. **b** The expression of LAMA4 and miR-539 in clinical specimens showed an inverse correlation between them; r^2^ = 0.2465, *p < 0.05, Pearson correlation analysis

To identify the microRNA that targets LAMA4, we queried several microRNA target databases, such as miRanda, TargetScan and miRBase, and predicted that probable related miRNAs bind directly to the 3′-UTR of LAMA4, including: miRNA-539, miRNA-217, miRNA-129-5p, miR-196b, miR-196a, miR-499-5p, miR-21, miR-590-5p, miR-300, miR-381, miR-125a-5p, miR-410, miR-543, miR-495, miR-33a, miR-33b, miR-197, miR-397-5p, miR-494, miR-19a, miR-19b and so on. Among these, miRNA-539 caught our attention, as it has been reported involved in several kinds of cancers but haven’t in breast cancer. We tested the expression of miRNA-539 in 40 pairs of TNBC and the adjacent normal tissues by the use of qPCR. The results showed miRNA-539 was decreased in TNBC tissues compared with the adjacent normal tissues (Fig. 4a, n = 40). We further analyzed the expression of LAMA4 and miR-539 in clinical specimens with Pearson correlation analysis, which showed an inverse correlation between them (Fig. 4b; r^2^ = 0.2465, p < 0.05).

### MiR-539 targets LAMA4 and miR-539 mimic could reduce the expression of LAMA4 both in miRNA and protein level

**Fig. 5:**
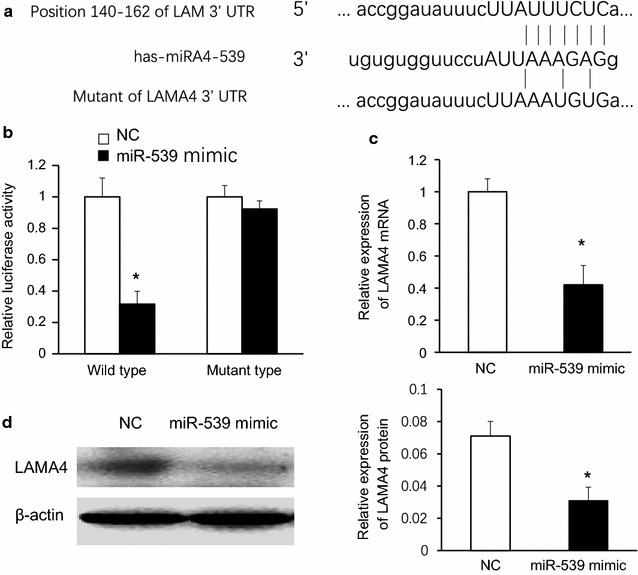
MiR-539 targets LAMA4 and miR-539 mimic could reduce the expression of LAMA4 both at miRNA and protein level. **a** The 3′UTR of LAMA4 contained the complementary sequences of miR-539. **b** Transfection of 293T cells with miR-539 mimic changed the LAMA4-regulated luciferase activity, *p < 0.05. **c** Upregulation of miR-539 reduced the expression of LAMA4 in mRNA level, *p < 0.05, N = 4, Student t test. **d** Upregulation of miR-539 reduced the expression of LAMA4 in protein level, *p < 0.05, N = 4, Student t test

To verify if miR-539 targets LAMA4, we researched the potential sequences in the 3′UTR of LAMA4 and found that the 3′UTR of LAMA4 contained the complementary sequences of miR-539 (Fig. 5a). Accordingly, we tested whether enhanced miR-539 expression could change the 3′UTR of LAMA4-regulated luciferase activity by dual luciferase assay. Results showed that transfection of 293T cells with miR-539 mimic changed the LAMA4 regulated luciferase activity, a significant decrease in the Rluc/Fluc ratio was found when co-transfected with miR-539 mimic in the wild type but not in mutant type of LAMA4 compared with negative control group (NC) (Fig. 5b). qPCR results demonstrated that upregulation of miR-539 notably reduced the expression of target gene (LAMA4) (Fig. 5c, N = 4 for each group, Student t test). Furthermore, Western blotting results also showed decreased LAMA4 (Fig. 5d) expression, suggesting that miR-539 targets to LAMA4 3′-UTR and inhibits its expression.

### Overexpression of miR-539 inhibits TNBC cancer cell proliferation, migration and invasion in vitro

**Fig. 6:**
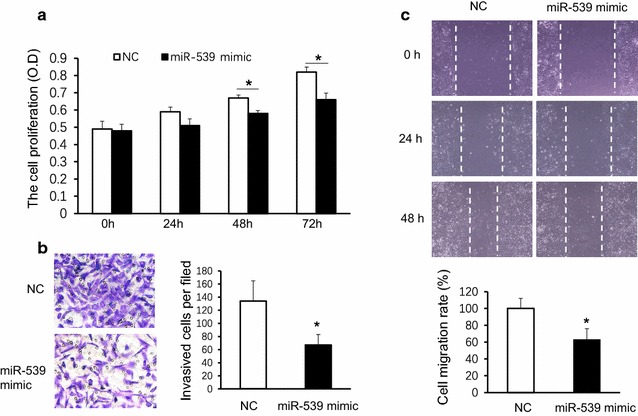
Overexpression of miR-539 inhibited TNBC cancer cell proliferation, migration and invasion in vitro. **a** The cell proliferation of miR-539 mimic transfected cells was inhibited at 48 h and lasted to 72 h, but not at 24 h; *p < 0.05, n = 4 for each group, Mann–Whitney test following Friedman ANOVA. **b** BT-549 cells transfected with miR-539 mimic inhibited the invasive ability, *p < 0.05, N = 4, Student t test. **c**. MiR-539 mimic could inhibit the cell migration rate, *p < 0.05, N = 4, Student t test

To study the role of miR-539 in the cancer development, BT-549 cells were transfected with miR-539 mimic or a scrambled miRNA (NC). The results showed that the cell proliferation of miR-539 mimic transfected cells was significantly inhibited at 48 h and lasted to 72 h, but neither at 24 h nor before compared either with the NC group cells (Fig. 6a, *p < 0.05, n = 4 for each group, Mann–Whitney test following Friedman ANOVA). To study if miR-539 was involved in tumor cells’ motility, cell migration was evaluated using wound healing and transwell assays. Results showed that BT-549 cells transfected with miR-539 mimic significantly inhibited the invasive ability, as the invasive cells per field in LV-LAMA4-shRNA3 group was 67 ± 16 while it was 134 ± 31 in NC group (Fig. 6b, *p < 0.05, N = 4 for each group, Student t test). Cell mobility was detected by the wound healing assay. At 24 and 48 h after wounding, the healing ability of miR-539-mimics-transfected BT-549 cells lagged behind the NC-transfected BT-549 cells. Images were captured at a magnification of × 10, and the columns represent the means ± SEMs of four independent experiments at 48 h. The results showed that miR-539 mimic could inhibited the cell migration rate (Fig. 6c, *p < 0.05, N = 4 for each group, Student t test).

## Discussion

Management of TNBC is a challenge to the clinician because of its aggressive behavior, poor outcome, and absence of targeted therapies. TNBCs have an increased propensity to metastasize and high rate of relapse, the majority of deaths from this disease are a result of distant disease [21]. A number of genes and proteins have been identified with altered expression in TNBC, like EGFR (epidermal growth factor receptor) [22, 23], PARPs [poly (ADP-ribose) polymerase] [24, 25], VEGF (vascular endothelial growth factor) [26], p53 [27, 28], TOP-2A (Topoisomerase 2 Alpha) [29] and so on. Till now, some of the markers have been tested for diagnostic and prognostic purposes. However, the vast majority of existing markers of tumor invasion and progression have not yet been put through a rigorous pre-clinical and clinical testing. Over time new targets that were specifically related to TNBC are being discovered for the treatment of this disease and is challenging [19].

In this study, we found LAMA4 was over-expressed in TNBC, the results were in consistent with a study by Ross et al. They found LAMA4 could promote cancer cell proliferation and increased LAMA4 expression marks the transition of human pre-malignant breast lesions to malignant carcinomas, and tumoral LAMA4 overexpression predicts reduced relapse-free survival in ER-negative patients [30]. But its role in the progression of TNBC is unknown. Through transfecting BT-549 cells with LV-LAMA4-shRNA in vitro, the expression of LAMA4 was reduced, and the cell proliferation, cell migration and invasion were inhibited. But the detail was unknown. As master regulators of gene expression, miRNAs are involved in modulating multiple cellular pathways, including cell proliferation, differentiation, and apoptosis, and thus may function as oncogenes or tumor suppressing genes [31, 32]. Recent studies have proved that the aberrant expression of miRNAs contribute to the proliferation, invasion or metastatic behavior of human breast cancer [33]. For example, HER2 and HER3 (erbB3), which are significantly correlated with decreased disease-specific survival in breast cancer patients, could be suppressed by miR-125a or miR-125b [34]. MiRNAs’ role in TNBC also has been discovered, for instance, the miR-146a and miR-26a were reported over-expressed in triple negative breast cancer and miR-10b and miR-153 were significantly associated to lymph node metastases occurrence in TNBC [35]. These results highlighted the role of miRNAs in breast cancer research. So we put our emphasis on miRNAs that modulated the expression of LAMA4, thus to find an effective intervention target to treat TNBC. MiR-539 is an important miRNA that has been reported to interfere progression of several tumors, it was found to be prognostic for distant metastasis-free survival in colon cancer [36]. Low expressions of miR-539 were found significantly associated with advanced TNM stage; metastasis; recurrence or reduced overall survival of osteosarcoma patients [37]. Besides, miR-539 was found to impair tumorigenesis of HCC cells in vivo, coupled with reduced expression of anti-apoptotic proteins Bcl-2 and Bcl-xL [38], but its role in TNBC is unknown. In this study, we found miR-539 was decreased in TNBC tissues and has an inverse correlation with the expression with LAMA4. MiR-539 mimic could reduce the expression of LAMA4 both at mRNA and protein level, so we identified LAMA4 was probably modulated by miR-539. But the mechanism needs further investigation.

## Conclusions

This study’s results indicate that LAMA4 was overexpressed in TNBC and inhibition the expression of which could suppress the TNBC cells’ migration and invasion. MiR-539 has an inverse correlation with the expression of LAMA4 and miR-539 mimic could inhibit TNBC cells’ ability of proliferation, migration and invasion. So LAMA4 might be the new target for treatment of TNBC. Further studies are needed to determine the molecular mechanisms of LAMA4 and the clinical value of miR-539 in TNBC.

## Abbreviations

LAMA4: laminin subunit alpha 4
TNBC: triple-negative breast cancer
IHC: immunohistochemical staining
miRNAs: MicroRNAs
UTR: untranslated region
PBS: phosphate-buffed saline
SDS: sodium dodecyl sulfate
BCA: bicinchoninic acid
FBS: fetal bovine serum
HEK-293T: Human Embryonic Kidney 293
NC: negative control
LV: lentiviral vector–derived
shRNA: short hairpin RNA
EGFR: epidermal growth factor receptor
PARPs: poly (ADP-ribose) polymerase
VEGF: vascular endothelial growth factor
TOP-2A: topoisomerase 2 alpha
